# Pinch-off syndrome–related port catheter migration requiring surgical extraction combined with coronary artery bypass grafting

**DOI:** 10.1093/jscr/rjag400

**Published:** 2026-05-26

**Authors:** Gözde Tekin, Mehmet Emre Elçi, Selen Öztürk, Hüseyin Uzandı, Mehmet Kızılay

**Affiliations:** Cardiovascular Surgery Department, Dr Siyami Ersek Thoracic and Cardiovascular Surgery Education and Research Hospital, Selimiye Mah, Tıbbiye Cad, No. 25, 34668 Uskudar, Istanbul, Turkey; Cardiovascular Surgery Department, Dr Siyami Ersek Thoracic and Cardiovascular Surgery Education and Research Hospital, Selimiye Mah, Tıbbiye Cad, No. 25, 34668 Uskudar, Istanbul, Turkey; Cardiovascular Surgery Department, Dr Siyami Ersek Thoracic and Cardiovascular Surgery Education and Research Hospital, Selimiye Mah, Tıbbiye Cad, No. 25, 34668 Uskudar, Istanbul, Turkey; Cardiovascular Surgery Department, Dr Siyami Ersek Thoracic and Cardiovascular Surgery Education and Research Hospital, Selimiye Mah, Tıbbiye Cad, No. 25, 34668 Uskudar, Istanbul, Turkey; Cardiovascular Surgery Department, Dr Siyami Ersek Thoracic and Cardiovascular Surgery Education and Research Hospital, Selimiye Mah, Tıbbiye Cad, No. 25, 34668 Uskudar, Istanbul, Turkey

**Keywords:** pinch-off syndrome, port catheter migration, catheter embolization, endovascular retrieval failure, surgical extraction

## Abstract

Pinch-off syndrome is a rare but potentially serious complication of subclavian venous port systems, resulting from mechanical compression between the clavicle and first rib and leading to catheter fracture and embolization. A 63-year-old man with lung cancer presented with a fractured port catheter that had migrated into the left main and lower lobe pulmonary arteries. Endovascular retrieval was attempted but was unsuccessful due to firm endothelial adhesion. Preoperative coronary angiography revealed concomitant significant coronary artery disease. The patient subsequently underwent successful surgical catheter extraction combined with coronary artery bypass grafting. This case illustrates a Grade 3 pinch-off syndrome and highlights the limitations of percutaneous techniques in chronically embedded catheters. It also demonstrates that a combined surgical approach can be a safe and effective strategy in selected patients.

## Introduction

Totally implantable venous access devices (TIVADs) are widely used in oncology patients requiring long-term chemotherapy and repeated vascular access. Although generally safe, these devices may be associated with mechanical complications such as catheter fracture, migration, and embolization, which can occasionally result in serious and potentially life-threatening events.

One of the most well-recognized mechanisms leading to catheter fracture is pinch-off syndrome (POS), which refers to mechanical compression of the catheter between the clavicle and the first rib following subclavian vein catheterization. The reported incidence of POS ranges from approximately 1% to 5% in patients with subclavian venous access devices. Repeated compression may lead to progressive catheter deformation, luminal narrowing, and eventual transection, allowing distal embolization of the catheter fragment into the heart or pulmonary arterial system.

In this report, we describe a case of Grade 3 POS with migration of a port catheter into the pulmonary arterial system. Endovascular retrieval was unsuccessful due to suspected endothelial adhesion, and the presence of significant coronary artery disease necessitated combined surgical catheter extraction and coronary artery bypass grafting (CABG). Written informed consent was obtained from the patient and the patient’s relatives for publication of this case report and accompanying images.

## Case presentation

A 63-year-old man with a history of lung carcinoma had a totally implantable venous access port inserted via the subclavian vein for chemotherapy administration. The device had been implanted 2 years earlier for the administration of eight cycles of chemotherapy.

**Figure 1:**
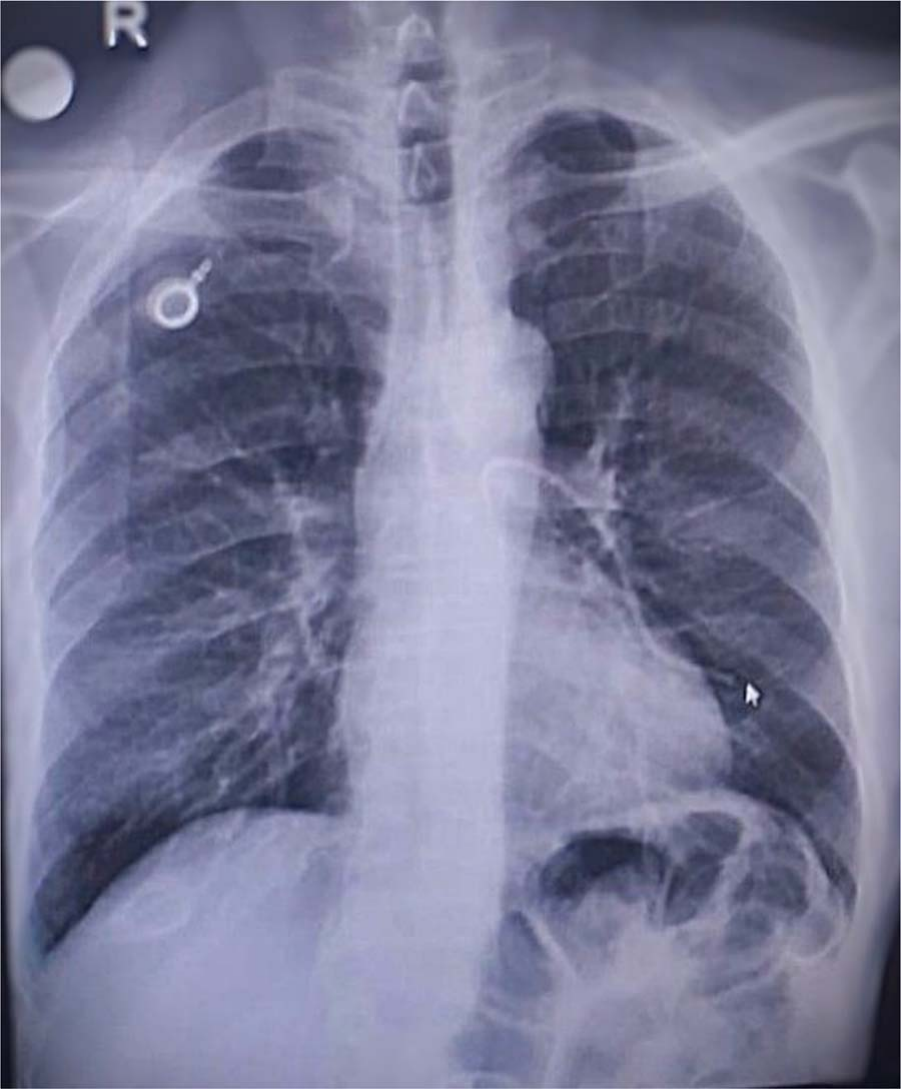
Chest imaging demonstrating complete migration of the fractured port catheter into the left main and lower lobe pulmonary arteries, consistent with Grade 3 pinch-off syndrome.

The patient presented with chest pain and dyspnea. Chest radiography revealed abnormal positioning of the catheter tip. Further imaging demonstrated that the port catheter had migrated into the left main and lower lobe pulmonary arteries (Fig. 1).

An endovascular retrieval attempt was performed via the right femoral venous approach using a loop snare device under fluoroscopic guidance. However, the catheter could not be mobilized due to firm adhesion to the vessel wall, suggesting long-standing endothelialization.

Because of the patient’s symptoms, coronary angiography was performed, which revealed significant stenoses in the left anterior descending (LAD) and right coronary artery (RCA). Based on these findings, the patient was scheduled for combined surgical catheter removal and CABG (Supplementary Video S1).

Through a median sternotomy, the left internal mammary artery (LIMA) and the saphenous vein graft were harvested in a standard fashion. Cardiopulmonary bypass was then established using aortic and bicaval cannulation. After initiation of cardiopulmonary bypass, the left pulmonary artery was explored, and the migrated catheter was identified within the vessel lumen. The catheter was found to be firmly adherent to the vessel wall and surrounded by fibrotic tissue, consistent with long-standing endothelialization. Careful dissection allowed complete removal of the foreign body without rupture or residual fragments.

**Figure 2:**
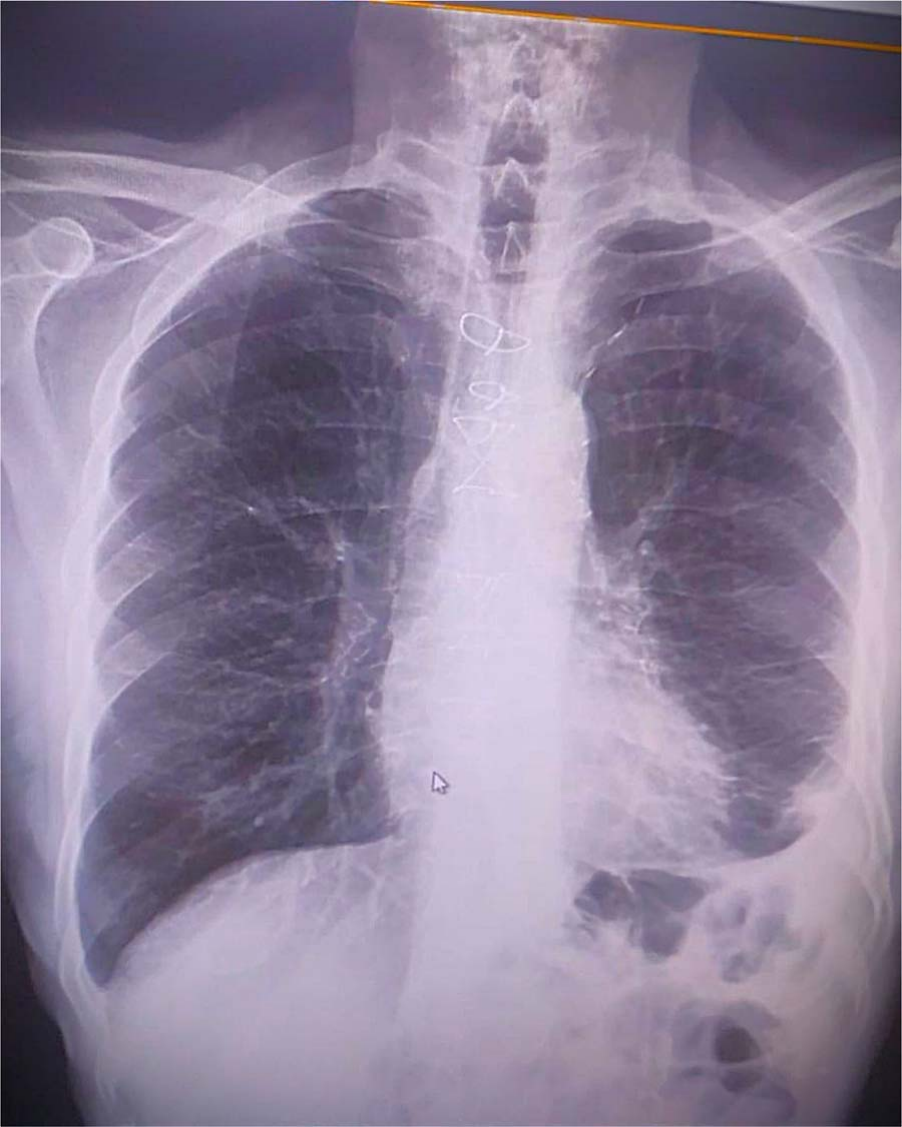
Postoperative chest imaging following successful surgical removal of the migrated catheter and completion of coronary artery bypass grafting, showing no residual foreign material.

The total cardiopulmonary bypass time was 154 min, with an aortic cross-clamp time of 82 min. Subsequently, two-vessel CABG was performed using the LIMA to the LAD artery (LIMA–LAD) and a saphenous vein graft to the right coronary artery (SVG–RCA). The patient was weaned from cardiopulmonary bypass without difficulty, and hemostasis was achieved before routine sternal closure. The patient was extubated at the 6th postoperative hour, transferred from the intensive care unit to the ward on postoperative day 1, and discharged on the 7th postoperative day without complications (Fig. 2).

## Discussion

Migration of TIVAD catheters into the pulmonary arterial system is a rare but recognized complication, usually associated with mechanical stress, improper fixation, or long-term catheter fatigue. In most cases, embolized catheter fragments can be successfully retrieved using endovascular techniques, particularly loop-snare systems, which are considered first-line due to their high success rate and minimally invasive nature [1–3]. Modified retrieval strategies, such as adjustable loop systems and the RAPID-HANDLE technique, have also been described [4].

Migration or fracture of totally implantable port catheters remains an uncommon but potentially serious complication. When catheter fragments remain intravascular for a prolonged period, endothelial adhesion may develop, making percutaneous retrieval challenging or impossible. In such situations, surgical removal becomes the definitive treatment [5–7]. Our case illustrates this scenario, in which the migrated catheter could not be retrieved endovascularly due to firm adhesion to the pulmonary arterial wall.

Another mechanism leading to catheter fracture and embolization is POS, first described by Aitken and Minton in 1984 [8]. It results from compression between the clavicle and first rib after subclavian catheterization, leading to deformation, narrowing, and eventual transection with distal embolization.

Hinke *et al*. classified POS into four grades:

1. Grade 0—normal;
2. Grade 1—slight deviation;
3. Grade 2—compression;
4. Grade 3—complete fracture with embolization [9].

Our case corresponds to Grade 3 POS, with the catheter fragment located in the pulmonary arteries. Similar cases highlight the importance of early recognition and intervention [10].

Although endovascular techniques are effective in most cases, prolonged intravascular retention may result in endothelialization, limiting success [3, 4, 11]. In such situations, surgical extraction is a safe and definitive option.

In this patient, surgery was preferred not only due to failed endovascular retrieval but also because of concomitant coronary artery disease. Combined catheter removal and CABG allowed definitive treatment in a single session.

This case emphasizes the importance of long-term surveillance of venous access devices. Early radiographic signs of POS should prompt intervention, and clinicians should be aware that prolonged catheter migration may limit the success of percutaneous techniques.

## Conclusion

Migration of a port catheter into the pulmonary artery is a rare but potentially life-threatening complication, most commonly associated with POS. Early recognition of catheter compression on chest imaging is essential to prevent catheter fracture and distal embolization. When endovascular retrieval fails due to endothelial adhesion, surgical extraction remains a safe and definitive treatment option. This case also demonstrates that concomitant cardiac procedures, such as CABG, can be safely performed during the same operation, allowing definitive management in a single surgical session.

## Supplementary Material

rjag400_Supplemental_Files
