# Supplementary material for: Pinch-off syndrome–related port catheter migration requiring surgical extraction combined with coronary artery bypass grafting
Source: J Surg Case Rep. 2026 May 26;2026(5):rjag400. doi: 10.1093/jscr/rjag400 (PMC13201266; doi:10.1093/jscr/rjag400)
Supplement: rjag400_Supplemental_Files [file rjag400_supplemental_files.zip › Supplemantary_Video_1_rjag400.docx]

**Supplemantary Video 1.** Coronary angiography demonstrating the migrated port catheter within the pulmonary arterial system prior to surgical removal.
